# Effect of Taijiquan assisted rehabilitation for breast cancer patients: A protocol for systematic review and meta-analysis

**DOI:** 10.1097/MD.0000000000025380

**Published:** 2021-04-02

**Authors:** Sihua Zhao, Rongna Lian, Ruinian Zhang, Fanghong Wang, Hao Chen, Run Wan

**Affiliations:** a Lanzhou University First Hospital Nursing Department (School of Nursing Lanzhou University); b The First Clinical Medical College of Lanzhou University; c General Surgery Department of The First Hospital of Lanzhou University; d Lanzhou University Second Hospital Oncology Center; e Lanzhou University Second Hospital Nursing Department, Lanzhou, China.

**Keywords:** breast cancer, comprehensive and alternative medicine, efficacy, rehabilitation, Taijiquan

## Abstract

**Background:**

Taijiquan, as a supplementary and alternative method, has attracted more and more attention in the treatment of breast cancer. But up to now, no systematic review has been performed to evaluate the efficacy of Taijiquan in the treatment of breast cancer. In this study, Cochrane systematic review method will be used to evaluate the effect of Taijiquan in the rehabilitation process of breast cancer patients after treatment.

**Methods:**

PubMed, Embase. com, the Cochrane Central Register of Controlled Trials (CENTRAL), Web of Science, China National Knowledge Infrastructure (CNKI), Wanfang, and SinoMed will be searched to identify relevant studies up to May 31, 2021. We will include randomized controlled trials (RCTs) of the application of Taijiquan in post-treatment breast cancer patients. We will use the Cochrane bias risk assessment tool to assess the quality of included RCTs. We will use Stata 13.0 to perform pairwise meta-analyses using the inverse variance method. Subgroup analyses and sensitivity analyses will be conducted to investigate the sources of heterogeneity.

**Results:**

The results of this study will be published in a peer-reviewed journal.

**Conclusion:**

This study will comprehensively evaluate the efficacy of Taijiquan in the rehabilitation treatment of breast cancer. The results of this study will provide high-quality evidence to support clinical practice and guidelines development.

## 1 Introduction

Breast cancer (BC) is the most common cancer in women all over the world and the second most common cause of cancer-related mortality.^[1,2]^ The overall incidence rate of breast cancer is increasing rapidly in the world, which is positively correlated with the level of regional development.^[3]^ Complementary and alternative medicine (CAM) is a group of medical and health care embodiment, practice and products different from traditional medical behavior, complementary to traditional medicine, and belongs to the category of general practice.^[4,5]^ Compared with the traditional model, CAM has the advantages of strong practicability, short treatment induction period, economic and practical, which can stimulate the rehabilitation potential and improve the overall quality of life of patients with breast cancer while carrying out traditional treatment.^[6,7]^ CAM has become the main treatment of pain, fatigue, dyspnea, and other symptoms in patients with advanced breast cancer.^[8,9]^ Taijiquan as a complementary alternative method in the treatment of breast cancer has attracted more and more attention.^[10]^ But up to now, no systematic review has been used to evaluate the efficacy of Taijiquan in the rehabilitation process of breast cancer patients after treatment. In this study, the Cochrane systematic review method will be used to evaluate the efficacy of Taijiquan in the rehabilitation treatment of breast cancer, to provide high-quality evidence to support guidelines development and clinical practice, and promote the development of personalized rehabilitation of breast cancer.

## 2 Data and methods

We will conduct and report this meta-analysis according to the Preferred Reporting Items for Systematic Reviews and Meta-Analyses (PRISMA) statement.^[11]^ The protocol of this study has been registered on the International Platform of Registered Systematic Review and Meta-Analysis Protocols (INPLASY, INPLASY202130010).

### 2.1 Inclusion and exclusion criteria

#### 2.1.1 Type of study

Randomized controlled trials (RCTs) about Tai Chi for breast cancer will be included. We will exclude research reviews, cross-sectional studies, conferences, observational studies, and case reports.

#### 2.1.2 Type of participant

Women over 18 years old with breast cancer confirmed by pathology or cytology have received traditional western medicine treatment for breast cancer, including surgery, chemotherapy, radiotherapy, and hormone therapy, regardless of race and nationality.

#### 2.1.3 Type of interventions

It can be any type of Tai Chi, such as simplified Taijiquan, simplified Yang's Taijiquan, 19-style simple Taijiquan, and 24-style Taijiquan. It is not limited by the frequency, time, place, and intensity of intervention.

#### 2.1.4 Types of controls

The control group should adopt one of the following treatment methods: no treatment, placebo, usual or standard care, health education, psychosocial therapy, and drug therapy.

#### 2.1.5 Types of outcome measure

Physical function, quality of life, social function, mental state, grip strength, elbow flexion function, elbow extension, social well-being, and emotional health.

#### 2.1.6 Exclusion criteria

1. patients who did not meet the diagnostic criteria of breast cancer;
2. lymphatic drainage after breast cancer surgery;
3. physical function condition was not suitable for Taijiquan therapy;
4. psychiatric disorders or taking psychotropic drugs with the definite clinical diagnosis;
5. patients receiving a blood transfusion or steroid treatment;
6. patients with the second type of solid tumor who were not cured at the same time; and
7. the life span is less than 6 months.

### 2.2 Retrieval strategy

We will search English databases: PubMed, Embase.com, the Cochrane Central Register of controlled trials (CENTRAL) and Web of Science, as well as Chinese databases: China National Knowledge Infrastructure (CNKI), Wanfang, and Sinomed. The key words will include: “breast cancer,” “breast tumor,” “breast cancer,” “breast cancer,” “breast tumor,” “breast cancer,” “phyllodes tumor,” “intraductal carcinoma,” “lobular carcinoma,” “Tai-ji,” “tai chi,” “tai ji quan,” “Taiji,” “Taijiquan,” “tai chi chuan,” “Tai-ji,” “TCC,” “Intention to Treat Analysis,” “Pragmatic Clinical Trials as Topic,” “Clinical Trials, Phase II,” “Clinical Trials, Phase III,” “Clinical Trials, Phase IV,” “Controlled Clinical Trials,” “Randomized Controlled Trials,” “Single-Blind Method,” “Double-Blind Method,” “random,” “blind,” “singleblind,” “doubleblind,” “trebleblind,” “tripleblind”. Taking PubMed as an example, the specific retrieval strategy is shown in Table 1.

**Table 1:** Search strategy of PubMed.

#1	“Breast Neoplasms”[Mesh] OR “Breast Carcinoma In Situ”[Mesh] OR “Breast Neoplasms, Male”[Mesh] OR “Carcinoma, Ductal, Breast”[Mesh] OR “Carcinoma, Lobular”[Mesh] OR “Inflammatory Breast Neoplasms”[Mesh] OR “Triple Negative Breast Neoplasms”[Mesh] OR “Unilateral Breast Neoplasms”[Mesh] OR breast neoplasm∗[Title/Abstract] OR breast tumor∗[Title/Abstract] OR breast carcinoma∗[Title/Abstract] OR breast cancer∗[Title/Abstract] OR breast tumour∗[Title/Abstract] OR mammary neoplasm∗[Title/Abstract] OR mammary tumor∗[Title/Abstract] OR mammary carcinoma∗[Title/Abstract] OR mammary cancer∗[Title/Abstract] OR mammary tumour∗[Title/Abstract] OR breast adenocarcinoma∗[Title/Abstract] OR breast carcinogenesis[Title/Abstract] OR breast sarcoma∗[Title/Abstract] OR phyllodes tumor∗[Title/Abstract] OR intraductal carcinoma∗[Title/Abstract] OR lobular carcinoma∗[Title/Abstract]
#2	“tai ji”[Mesh] OR “Tai-ji”[Title/Abstract] OR “tai chi”[Title/Abstract] OR “tai ji quan”[Title/Abstract] OR “Taiji”[Title/Abstract] OR “Taijiquan”[Title/Abstract] OR “tai chi chuan”[Title/Abstract] OR “Tai-ji”[Title/Abstract] OR “TCC”[Title/Abstract]
#3	“Clinical Trials, Phase II as Topic”[Mesh] OR “Clinical Trials, Phase III as Topic”[Mesh] OR “Clinical Trials, Phase IV as Topic”[Mesh] OR “Controlled Clinical Trials as Topic”[Mesh] OR “Randomized Controlled Trials as Topic”[Mesh] OR “Intention to Treat Analysis”[Mesh] OR “Pragmatic Clinical Trials as Topic”[Mesh] OR “Clinical Trials, Phase II”[Publication Type] OR “Clinical Trials, Phase III”[Publication Type] OR “Clinical Trials, Phase IV”[Publication Type] OR “Controlled Clinical Trials”[Publication Type] OR “Randomized Controlled Trials”[Publication Type] OR “Pragmatic Clinical Trials as Topic”[Publication Type] OR “Single-Blind Method”[Mesh] OR “Double-Blind Method”[Mesh] OR random∗[Title/Abstract] OR blind∗[Title/Abstract] OR singleblind∗[Title/Abstract] OR doubleblind∗[Title/Abstract] OR trebleblind∗[Title/Abstract] OR tripleblind∗[Title/Abstract]
#4	#1 AND #2 AND #3

### 2.3 Literature screening and data extraction

Two reviewers will independently screen the literature, extract the data, and cross-check the data. In case of disagreement, a third party will be consulted to assist in judgment, and the author will be contacted to supplement the missing data if possible. In the process of literature selection, we will first read the titles and abstracts. After excluding the unrelated literatures, we will further read the full text to determine whether they are included. Data extraction included: author, publication time, randomization method, grouping and sample size, age and sex of patients, intervention method (operation name, course of treatment), baseline comparison, distribution, whether to use the blind method, the outcome of interest, and follow-up time.

### 2.4 Risk of bias assessment of included studies

Two reviewers will assess the risk of bias of included RCTs using the “Cochrane bias risk assessment tool.”^[12]^ The evaluation items include:

1. random sequence generation (selection bias).
2. allocation concealment (selection bias).
3. blinding of participants and personnel (performance bias).
4. blinding of outcome assessment (detection bias).
5. incomplete outcome data (attrition bias).
6. selective reporting (reporting bias).
7. other sources of bias (other bias).

Each item will be judged as low risk, high risk, and unclear risk.

### 2.5 Statistical analysis

#### 2.5.1 Data synthesis

We will use Stata (13.0; Stata Corporation, College Station, TX) for pooling data and statistical analysis. We will conduct a meta-analysis using the inverse variance method to compute relative risks (RRs) and their 95% confidence interval (CI) for dichotomous outcomes and mean difference (MD) and 95%CI for continuous outcomes. The statistical level of significance will be set at *P* < .05.

#### 2.5.2 Assessment of heterogeneity

Chi^2^ test will be used to analyze the statistical heterogeneity of the results, and *P* value and *I*^*2*^ will be used to quantitatively judge the heterogeneity. If the homogeneity of the included studies is low (*P* > .1 and *I*^*2*^ < 50%), the fixed-effect model will be used for meta-analysis; if there is heterogeneity between the included studies (*P* < .1 and *I*^*2*^ ≥ 50%), the source of heterogeneity will be further analyzed. After excluding the influence of obvious clinical heterogeneity, the random effect model will be used for meta-analysis. Significant clinical heterogeneity will be explored by subgroup analysis and sensitivity analysis.

#### 2.5.3 Subgroup analyses and meta-regression analyses

Univariate meta-regression analysis will be performed on the within-study factors (time, sample size, tumor pathological stage, previous treatment of breast cancer, intervention group scheme, intervention time) and between study factors (mean age, race) respectively to screen out the important factors leading to heterogeneity. Subgroup analysis will be performed on these significant factors.

#### 2.5.4 Sensitivity analysis

We will perform sensitivity analyses by excluding low-quality studies to assess the robustness of our conclusions.

#### 2.5.5 Publication bias

The publication bias will be explored using the funnel plot and Egger test for outcomes with studies no less than 10.

### 2.6 Certainty of evidence

We will create a “Summary of findings” table presenting our primary and secondary outcomes using the GRADEpro Guideline Development Tool (GDT) software.^[13]^ We will use the 5 Grading of Recommendations Assessment, Development, and Evaluation (GRADE) considerations (risk of bias, inconsistency, imprecision, indirectness, and publication bias) to assess the quality of the body of evidence for each meta-analysis.^[14–16]^ We will rate the quality of evidence as high, moderate, low, or very low, and will justify decisions to downgrade or upgrade the quality of the evidence using footnotes where necessary.

## 3 Result

### 3.1 Screening results

**Figure 1:**
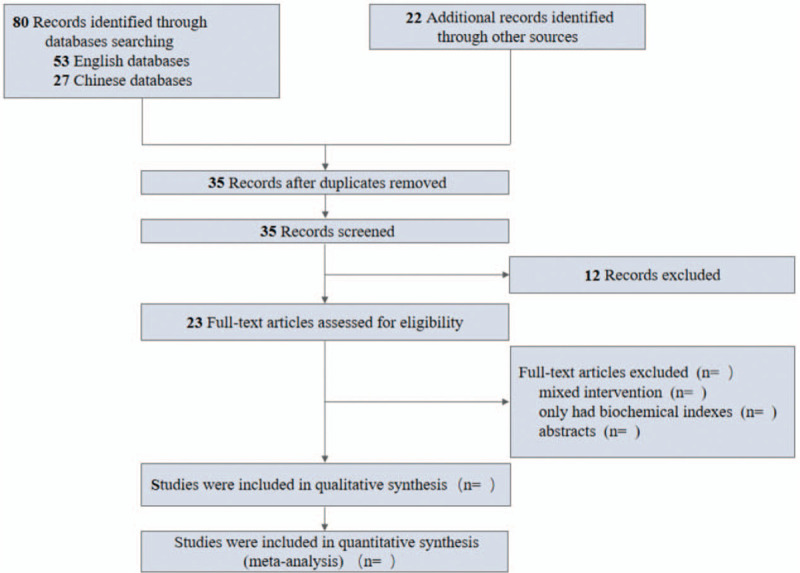
Flow chart of literature screening.

We conducted a pilot literature search, and a total of 102 articles were retrieved, and 35 articles were obtained by endnote. By looking at the title and abstract of the literature, 12 unrelated literatures were excluded. We will show the screening process in the prism flow chart (Fig. 1).

### 3.2 General characteristics and quality of studies

We presented characteristics of some included studies in Table 2. All RCTs are in English,^[17–25]^ publishing between 2006 and 2015. The details are shown in Table 2.

**Table 2:** Characteristics of partially included studies.

				Sample		Age				Taiji	
First author	Year	Country	Language	Taiji	Control	Taiji	Control	Tumor staging	Previous treatment	Treatment cycle	Frequency
Mustian et al^[17]^	2008	USA	English	11	10	52 (33–78)	I–IIIb	surgery+radiation, chemotherapy, hormone therapy	12 weeks	3 times a week for 60 minutes	
Sprod et al^[18]^	2012	USA	English	9	10	average 53	average 53	I–IIIb	surgery, radiation, chemotherapy, hormone therapy	12 weeks	3 times a week for 60 minutes
Robins et al^[19]^	2013	USA	English	37	36	50	50	I–IIIa	chemotherapy	42 weeks	3 times a week for 60 minutes
Janelsins et al^[20]^	2011	USA	English	9	10	average 54.3	average 52.7	I–IIIb	surgery, radiotherapy, chemotherapy	12 weeks	3 times a week for 60 minutes
Mustian et al^[21]^	2004	USA	English	11	10	52 (33–78)	0-III	surgery, radiation, chemotherapy, hormone therapy	12 weeks	3 times a week for 60 minutes	
Peppone et al^[22]^	2010	USA	English	7	9	53.8	52.9	I–IIIb	surgery, hormone therapy	12 weeks	Twice a day for 20 to 30 minutes
Larkey et al^[23]^	2015	USA	English	42	42	57.7 (8.94)	59.8 (8.93)	0-III	surgery, radiation, and/or chemotherapy	12weeks	60 minutes twice a week, followed by 60 minutes once a week
Irwin et al^[24]^	2014	USA	English	45	45	59.6 (7.9)	60.0 (9.3)	Not mentioned	surgery, radiation, and/or chemotherapy	3 months	2 hours a week
Mustian et al^[25]^	2006	USA	English	11	10	52 (33–78)	I–IIIb	surgery, radiation, chemotherapy, hormone therapy	12 weeks	3 times a week for 60 minutes	

## 4 Discussion

CAM has become the main treatment of pain, fatigue, dyspnea, and other symptoms in patients with advanced breast cancer.^[8,9]^ At present, the most effective method for the treatment of breast cancer is the comprehensive treatment based on radical surgery. We believe the results of our study will provide high-quality evidence to support clinical practice.

## Author contributions

**Conceptualization:** Sihua Zhao, Rongna Lian, Ruinian Zhang, Fanghong Wang, Hao Chen, Run Wan.

**Funding acquisition:** Fanghong Wang.

**Methodology:** Sihua Zhao, Rongna Lian, Ruinian Zhang, Fanghong Wang, Hao Chen, Run Wan.

**Writing – original draft:** Sihua Zhao, Rongna Lian.

**Writing – review & editing:** Sihua Zhao, Rongna Lian, Ruinian Zhang, Fanghong Wang, Hao Chen, Run Wan.
